# MicroRNA-150 suppresses cell proliferation and metastasis in hepatocellular carcinoma by inhibiting the GAB1-ERK axis

**DOI:** 10.18632/oncotarget.7292

**Published:** 2016-02-09

**Authors:** Wei Sun, Zhuochao Zhang, Jianlin Wang, Runze Shang, Liang Zhou, Xing Wang, Juanli Duan, Bai Ruan, Yuan Gao, Bin Dai, Shibin Qu, Wei Liu, Rui Ding, Lin Wang, Desheng Wang, Kefeng Dou

**Affiliations:** ^1^ Department of Hepatobiliary Surgery, Xijing Hospital, The Fourth Military Medical University, Xi'an, Shaanxi, China; ^2^ Department of General Surgery, The 155th Central Hospital of PLA, Kaifeng, Henan, China; ^3^ Department of Hepatobiliary Surgery, The 224th Hospital of PLA, Jiamusi, Heilongjiang, China

**Keywords:** hepatocellular carcinoma, miR-150, GAB1, epithelial mesenchymal transition, metastasis

## Abstract

MicroRNA-150 (miR-150) is frequently dysregulated in cancer and is involved in carcinogenesis and cancer progression. In this study, we found that miR-150 was significantly downregulated in hepatocellular carcinoma (HCC) tissues compared to adjacent noncancerous tissues. Low levels of miR-150 were significantly associated with worse clinicopathological characteristics and a poor prognosis for patients with HCC. miR-150 overexpression inhibited cell proliferation, migration and invasion *in vitro* and tumor growth and metastasis *in vivo*. Further experiments indicated that Grb2-associated binding protein 1 (GAB1) was a direct target of miR-150 in HCC cells. In addition, GAB1 expression was increased in HCC tissues and inversely correlated with miR-150 levels. Knockdown of GAB1 mimicked the tumor-suppressive effects of miR-150 overexpression on HCC cells, whereas restoration of GAB1 expression partially abolished the inhibitory effects. Moreover, miR-150 overexpression decreased GAB1 expression, subsequently downregulated phospho-ERK1/2 and suppressed epithelial-mesenchymal-transition (EMT). These effects caused by miR-150 overexpression were alleviated by exogenous GAB1 expression. Taken together, this study demonstrates that miR-150 may be useful as a prognostic marker and that the identified miR-150-GAB1-ERK axis is a potential therapeutic target for HCC.

## INTRODUCTION

Hepatocellular carcinoma (HCC) is the sixth most common cancer and the third leading cause of cancer-related mortality worldwide [1]. More than 110,000 patients die from HCC every year in China [2]. Despite the clinical implementation of numerous therapeutic strategies, patients with HCC have a poor prognosis due to intrahepatic recurrence and tumor metastasis [3, 4]. Although substantial advances have been made in the functional genomics of HCC development, a better understanding of the molecular events underlying the recurrence and metastasis of HCC is still very important for the development of effective targeted therapies.

MicroRNAs (miRNAs) are a class of endogenously expressed small non-coding RNAs that regulate gene expression by binding to the 3′ untranslated region (3′-UTR) of their target mRNAs, resulting in either degradation or repression of protein translation [5, 6]. Accumulating evidence suggests that miRNAs play critical roles in a range of biological processes, including cell proliferation, apoptosis, differentiation, metabolism, maturation, invasion and metastasis, by regulating critical tumor suppressors or oncogenes [7–9]. Aberrant expression of miRNAs has been shown to contribute to the development and progression of many types of human tumors, including HCC [10–13].

MicroRNA-150 (miR-150) was initially identified as a key miRNA in hematopoietic and immune cells [14]. Recent studies have demonstrated that miR-150 also participates in various cell functions in different types of tumors. For example, miR-150 expression is downregulated in colorectal cancer compared to paired non-cancerous tissue and acts as a tumor suppressor by targeting c-Myb [15, 16]. Wang et al. reported that miR-150 suppresses colorectal cancer cell migration and invasion by directly targeting MUC4 [17]. In human epithelial ovarian cancer (EOC), miR-150 downregulation was significantly associated with aggressive clinicopathological features of patients with EOC, and miR-150 may function as a tumor suppressor by directly and negatively regulating ZEB1 [18]. miR-150 is also overexpressed in many types of tumors, such as lung cancer [19], breast cancer [20] and gastric cancer [21]. These contrasting results indicate that miR-150 may have diverse roles in different types of cancers. However, to our knowledge, the roles and mechanism of miR-150 in the regulation of HCC progression have not been completely elucidated. In this study, we first systematically analyzed the association of miR-150 expression with the clinicopathological characteristics and prognosis of patients with HCC. Based on these results, we investigated the biological functions and molecular mechanisms of miR-150 in the development of HCC. We report that miR-150 expression is downregulated in HCC tissues and acts as a tumor suppressor by inhibiting the GAB1-ERK axis in HCC.

## RESULTS

### miR-150 expression is downregulated in HCC tissues and associated with clinicopathological characteristics and prognosis in patients with HCC

**Figure 1:**
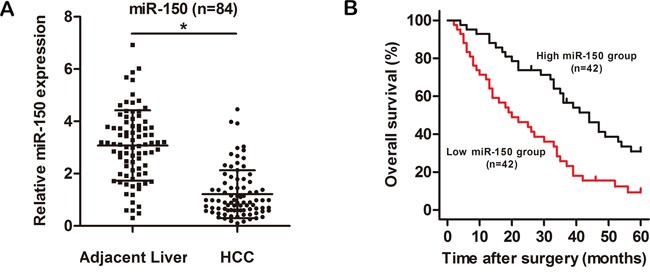
miR-150 is significantly downregulated in HCC tissues **A.** The relative expression level of miR-150 in HCC tissues (n=84) and in matched adjacent noncancerous liver tissues (n=84). **B.** Kaplan-Meier analysis of the overall survival of patients with HCC with high (n=42) and low (n=42) levels of miR-150 expression. * *P*<0.05.

The levels of miR-150 in 84 HCC tissues and adjacent noncancerous liver tissues were quantitated by qRT-PCR. As shown in Figure 1A, miR-150 expression was significantly lower in HCC tissues than in adjacent noncancerous tissues (*P*<0.05). Next, we investigated the clinicopathological significance of miR-150 in patients with HCC. The relationships between miR-150 expression and various clinicopathological characteristics of patients with HCC are shown in Table 1. Low miR-150 expression was significantly correlated with tumor size (*P*<0.05), venous invasion (*P*<0.05) and metastasis (*P*<0.05) but not with other clinicopathological characteristics, such as sex, age, liver cirrhosis, tumor number, differentiation and TNM stage, in patients with HCC.

**Table 1:** Correlations between miR-150 expression and clinicopathological characteristics in 84 patients with HCC

Characteristics	n	miR-150 expression		*p*-values
		High	Low	
All case	84	42	42	
Gender				
Male	64	30 (46.9%)	34 (53.1%)	0.306
Female	20	12 (60.0%)	8 (40.0%)	
Age				
≥50	43	23 (53.5%)	20 (46.5%)	0.513
<50	41	19 (46.3%)	22 (53.7%)	
Liver cirrhosis				
Presence	60	27 (45.0%)	33 (55.0%)	0.147
Absence	24	15 (62.5%)	9 (37.5%)	
Tumor size (cm)				
>5	41	16 (39.0%)	25 (61.0%)	0.049*
≤5	43	26 (60.5%)	17 (39.5%)	
Tumor number				
Single	61	32 (52.5%)	29 (47.5%)	0.463
Multiple	23	10 (43.5%)	13 (56.5%)	
Differentiation				
High	59	33 (55.9%)	26 (44.1%)	0.095
Low	25	9 (36.0%)	16 (64.0%)	
Venous invasion				
Yes	31	11 (35.5%)	20 (64.5%)	0.042*
No	53	31 (58.5%)	22 (41.5%)	
Metastasis				
Yes	19	5 (26.3%)	14 (73.7%)	0.019*
No	65	37 (56.9%)	28 (43.1%)	
TNM stage				
I + II	46	27 (58.7%)	19 (41.3%)	0.079
III + IV	38	15 (39.5%)	23 (60.5%)	

* Statistically significant difference; TNM: T, tumor; N, lymph node; M, distant metastasis.

The Kaplan-Meier survival analysis revealed that patients with low miR-150 expression had a shorter overall survival time than those with high miR-150 expression (Figure 1B, *P*<0.05). Univariate Cox regression analysis was performed to assess the prognostic value of miR-150. As shown in Table 2, miR-150 expression (*P*<0.05), venous invasion (*P*<0.05), metastasis (*P*<0.05) and TNM stage (*P*<0.05) were significantly associated with the overall survival of patients with HCC. Multivariate Cox regression analysis demonstrated that miR-150 expression (*P*<0.05), metastasis (*P*<0.05) and TNM stage (*P*<0.05) were independent prognostic factors of survival of patients with HCC (Table 2, lower panel). Taken together, these results indicate that low expression of miR-150 is involved in HCC progression.

**Table 2:** Univariate and multivariate Cox regression analyses of overall survival in 84 patients with HCC

Tumor characteristic	HR (95% CI)	*p*-value
Univariate Analysis (Cox: Enter)		
Gender(Female / Male)	1.158(0.640-2.095)	0.628
Age(<50 / ≥50)	1.036(0.634-1.691)	0.889
Liver cirrhosis(Absence / Presence)	1.494(0.848-2.633)	0.165
Tumor size (≤5cm / >5cm)	1.543(0.942-2.527)	0.085
Tumor number(Single / Multiple)	1.588(0.926-2.722)	0.093
Differentiation(High / Low)	1.587(0.939-2.681)	0.084
Venous invasion(− / +)	6.880(3.912-12.102)	<0.001*
Metastasis(− / +)	10.115(4.949-20.671)	<0.001*
TNM stage(I, II / III, IV)	6.427(3.642-11.343)	<0.001*
miR-150 (Low / High)	2.363(1.429-3.907)	0.001*
Multivariate Analysis (Cox: Forward LR)		
Metastasis(− / +)	3.413(1.593-7.315)	0.002*
TNM stage(I, II / III, IV)	4.888(2.562-9.326)	<0.001*
miR-150 (Low / High)	2.048(1.209-3.468)	0.008*

* Statistically significant difference; CI: confidence interval.

### miR-150 suppresses the proliferation, migration and invasion of HCC cells *in vitro*

**Figure 2:**
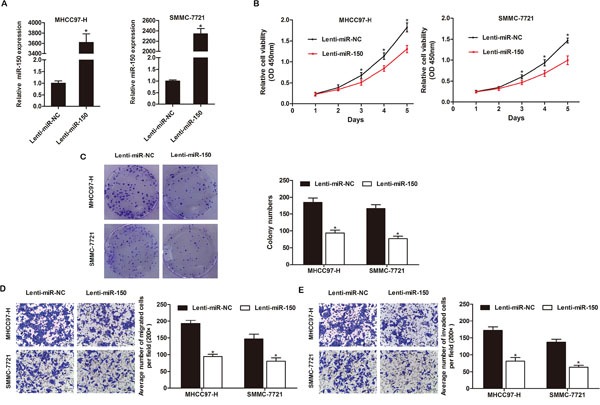
miR-150 inhibits HCC cell proliferation, colony formation, migration and invasion *in vitro* **A.** Expression of miR-150 in MHCC97-H and SMMC-7721 cells was confirmed by qRT-PCR. **B.** miR-150 overexpression significantly inhibited the growth rate of both MHCC97-H and SMMC-7721 cells as demonstrated by CCK-8 proliferation assay. **C.** Ectopic miR-150 expression significantly reduced colony formation by MHCC97-H and SMMC-7721 cells. miR-150 overexpression significantly inhibited the migration **D.** and invasion **E.** of MHCC97-H and SMMC-7721 cells. Migrated and invaded cells were counted in 5 randomly selected areas under a 200× microscope field. *P<0.05.

First, we detected the expression levels of miR-150 in the HCC cell lines by qRT-PCR. miR-150 was significantly lower in HCC cells compared to the normal human hepatocyte cell line HL-7702 (Supplementary Figure 1). To investigate the role of miR-150 in HCC progression, we transfected the HCC cell lines MHCC97-H and SMMC-7721 with a lentiviral plasmid expressing miR-150 (Lenti-miR-150) or a lentiviral control plasmid (Lenti-miR-NC) to stably overexpress miR-150 or negative control, respectively. qRT-PCR showed that miR-150 was significantly upregulated in the HCC cells transfected with Lenti-miR-150 (Figure 2A, *P*<0.05).

A CCK-8 assay was performed to quantitate the proliferation of the transfected HCC cells. miR-150 overexpression significantly inhibited the proliferation of MHCC97-H and SMMC-7721 cells compared to the Lenti-miR-NC-expressing cells (Figure 2B, *P*<0.05). Furthermore, a colony formation assay showed that miR-150 overexpression led to a significant reduction in colony counts compared to Lenti-miR-NC-expressing MHCC97-H and SMMC-7721 cells (Figure 2C, *P*<0.05).

Moreover, we explored the impact of miR-150 expression on the migration and invasion of HCC cells in Transwell assays. We observed that miR-150 overexpression suppressed the migration and invasion of MHCC97-H and SMMC-7721 cells. The migration rates of Lenti-miR-150-expressing MHCC97-H and SMMC-7721 cells were 50.95% and 45.12% lower, respectively, than the rates of Lenti-miR-NC-expressing MHCC97-H and SMMC-7721 cells (Figure 2D, *P*<0.05). Similarly, the invasion rates of Lenti-miR-150-expressing MHCC97H and SMMC-7721 cells were 52.52% and 53.64% lower, respectively, than the rates of Lenti-miR-NC-expressing MHCC97-H and SMMC-7721 cells (Figure 2E, *P*<0.05).

### miR-150 overexpression inhibits the tumor growth and metastasis of HCC cells *in vivo*

**Figure 3:**
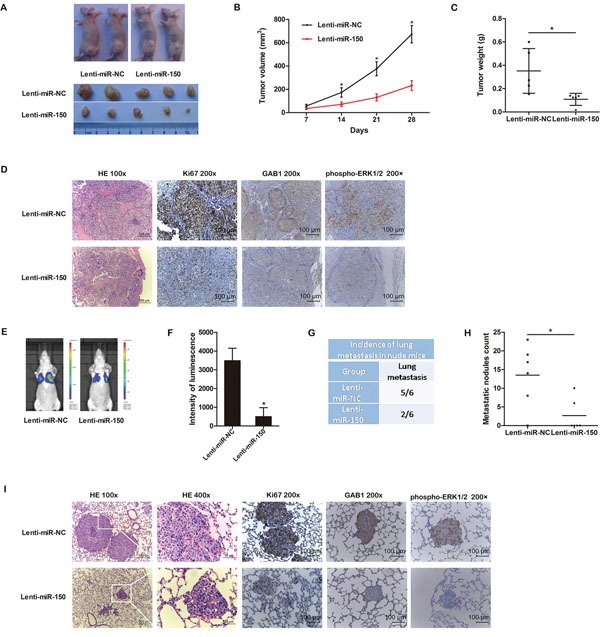
miR-150 suppresses tumor growth and metastasis *in vivo* **A.** Transduced MHCC97-H cells were injected subcutaneously into nude mice (n=5). After 28 days, the mice were euthanized, and the tumors were excised. Representative tumors are shown. **B.** Growth curve of tumor volumes. **C.** Tumor weight. **D.** Representative H&E and immunohistochemical staining for Ki-67, GAB1 and phospho-ERK1/2 in tumors are shown. **E.** Representative bioluminescence images 35 days after injection are shown. **F.** The luminescence intensity of lung metastases from MHCC97-H-luc-miR-150 cells was weaker than that of lung metastases from MHCC97-H-luc-miR-NC cells. **G.** miR-150 reduced the incidence of lung metastases. **H.** miR-150 overexpression significantly reduced the number of lung metastatic nodules. **I.** Representative H&E and immunohistochemical staining for Ki-67, GAB1 and phospho-ERK1/2 in lung metastatic nodules are shown. *P<0.05.

The effects of miR-150 overexpression on the growth of HCC cells were further confirmed by examining tumorigenicity *in vivo*. Immunodeficient BALB/C mice were subcutaneously injected with MHCC97-H cells that had been previously transfected with Lenti-miR-150 or Lenti-miR-NC. After 28 days, the volume and weight of the tumors generated from Lenti-miR-150-expressing MHCC97-H cells were significantly smaller than those generated by Lenti-miR-NC-expressing MHCC97-H cells (Figure 3A–3C, Supplementary Figure 2, *P*<0.05). Throughout the tumorigenic period, the tumors formed from Lenti-miR-150-expressing MHCC97-H cells grew significantly slower than those formed from Lenti-miR-NC-expressing MHCC97-H cells (Figure 3B, *P*<0.05).

To study the effects of miR-150 expression on tumor metastasis *in vivo*, we transfected luciferase-labeled MHCC97-H cells with Lenti-miR-150 or Lenti-miR-NC and injected the cells into nude mice intravenously. After 35 days, bioluminescent imaging showed that two of the six mice injected with Lenti-miR-150-expressing MHCC97-H-luc cells exhibited lung metastases, whereas five of the six mice injected with Lenti-miR-NC-expressing MHCC97-H-luc cells displayed pulmonary metastatic nodules (Figure 3E, 3G). The bioluminescence of the lung metastases from MHCC97-H-luc-miR-150 cells was weaker than that of the lung metastases from MHCC97-H-luc-miR-NC cells (Figure 3F, *P*<0.05). Moreover, upregulation of miR-150 decreased the number of metastatic lung nodules (Figure 3H, *P*<0.05). Representative hematoxylin and eosin (H&E) staining of metastatic lung nodules are shown in Figure 3I.

In addition, we performed immunohistochemistry to detect Ki-67 in tumors and lung metastatic nodules. The tumors and lung metastatic nodules from MHCC97-H-miR-150 cells displayed much weaker staining of Ki-67 than those from MHCC97-H-miR-NC cells (Figure 3D, 3I), indicating that miR-150 overexpression inhibited MHCC97-H cell proliferation *in vivo*.

### GAB1 is a direct target of miR-150

**Figure 4:**
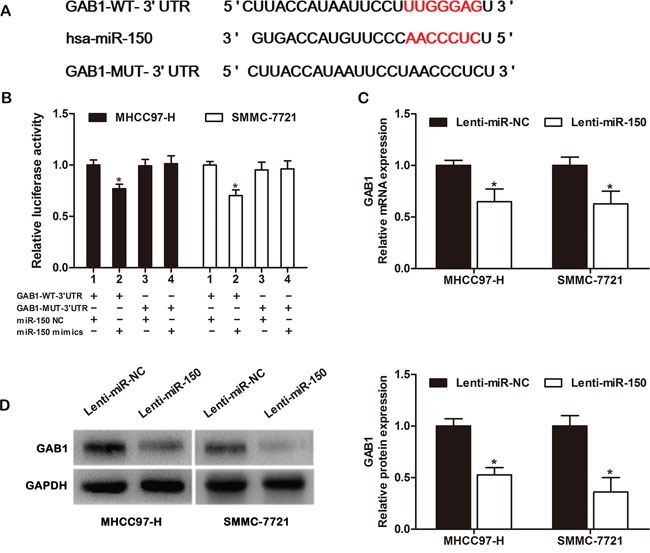
GAB1 is a direct target of miR-150 in HCC cells **A.** Predicted miR-150 binding site in the 3′-UTR region of GAB1. A mutated miR-150 binding site was generated in the complementary site for the seed region of miR-150. **B.** A miR-150 mimic or negative control and a luciferase vector encoding the wild-type or mutant GAB1 3′-UTR region were introduced into MHCC97-H and SMMC-7721 cells, and the relative luciferase activity was measured. **C.** Quantitation of GAB1 mRNA levels in MHCC97-H and SMMC-7721 cells by qRT-PCR after transfection with Lenti-miR-150 or Lenti-miR-NC. **D.** Detection of GAB1 protein in MHCC97-H and SMMC-7721 cells by western blot analysis after transfection with Lenti-miR-150 or Lenti-miR-NC. *P<0.05.

To understand the molecular mechanisms by which miR-150 suppresses HCC progression, we used bioinformatics tools (TargetScan, miRanda, and miRWalk) to predict putative target genes. Among the candidates, GAB1, an important oncogene, was chosen for further analysis. A predicted binding site for miR-150 was identified in the 3′-UTR of GAB1 mRNA (Figure 4A). To determine whether GAB1 is a direct target of miR-150, we constructed luciferase reporter plasmids encoding the wild-type 3′-UTR region of GAB1 (GAB1-WT-3′-UTR) or a mutated GAB1 3′-UTR region (GAB1-MUT-3′-UTR) (Figure 4A). As shown in Figure 4B, miR-150 significantly decreased the relative luciferase activity of the GAB1-WT-3′-UTR reporter in both MHCC97-H and SMMC-7721 cells (*P*<0.05), whereas the activity of the GAB1-MUT-3′-UTR reporter was not affected by miR-150. In addition, we performed qRT-PCR and western blot analysis to further investigate the interaction between miR-150 and GAB1. qRT-PCR showed that miR-150 overexpression significantly decreased GAB1 mRNA expression in both MHCC97-H and SMMC-7721 cells (Figure 4C, *P*<0.05). Western blot analysis revealed that miR-150 overexpression markedly lowered the levels of GAB1 protein in HCC cells (Figure 4D, *P*<0.05).

### miR-150 suppresses the proliferation, migration and invasion of HCC cells by targeting GAB1

**Figure 5:**
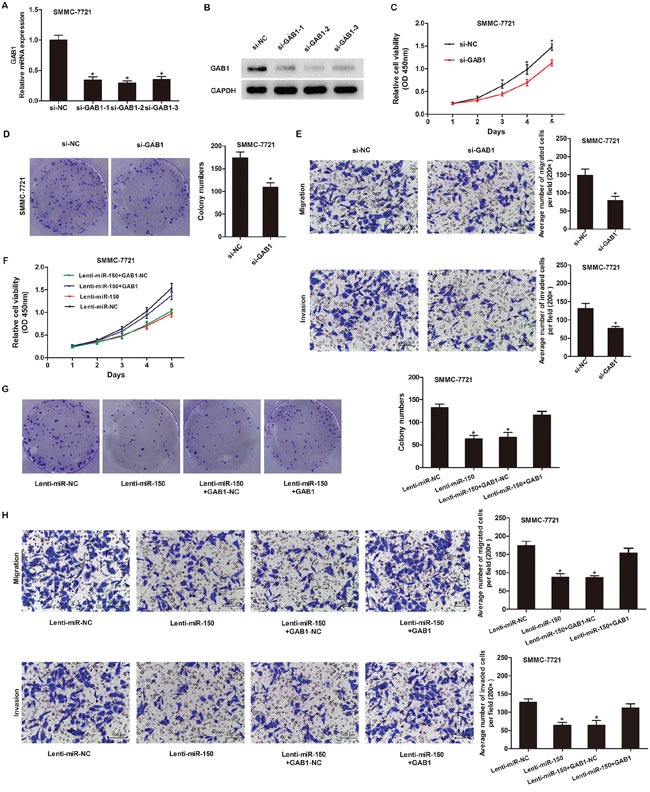
miR-150 inhibits HCC cell proliferation, migration and invasion by targeting GAB1 **A.** Quantitation of GAB1 mRNA levels in SMMC-7721 cells by qRT-PCR after transfection with 3 different siRNAs targeting GAB1 (si-GAB1-1, si-GAB1-2 and si-GAB1-3) or a negative control siRNA (si-NC). **B.** Detection of GAB1 protein in SMMC-7721 cells by western blot analysis after transfection with GAB1 siRNAs or si-NC. **C-E.** GAB1 knockdown inhibited SMMC-7721 cell proliferation, colony formation, migration and invasion as demonstrated by CCK-8 proliferation assay (C), colony formation assay (D) and Transwell assay (E). **F-H.** GAB1 reintroduction into SMMC-7721 cells partially rescued the miR-150-mediated inhibition of cell proliferation, colony formation, migration and invasion as determined by CCK-8 proliferation assay (F), colony formation assay (G) and Transwell assay (H). Migrated and invaded cells were counted in 5 randomly selected areas under a 200× microscope field. *P<0.05.

To investigate whether miR-150 exerted its effects by regulating GAB1 in HCC cell lines, we transfected SMMC-7721 cells with GAB1 siRNAs to knockdown endogenous GAB1 expression. qRT-PCR and western blot analysis were performed to confirm reduction of GAB1 levels (Figure 5A, 5B). All 3 GAB1-siRNAs reduced GAB1 expression, and the most effective siRNA (si-GAB1-2) was used for additional experiments. GAB1 knockdown significantly inhibited the proliferation, migration and invasion of SMMC-7721 cells (Figure 5C–5E, *P*<0.05) in a manner similar to miR-150 overexpression in these cells. Furthermore, the inhibitory effects of miR-150 on HCC cells were partially counteracted by restoring GAB1 expression (Figure 5F–5H, *P*<0.05).

### GAB1 expression is inversely correlated with miR-150 expression in patients with HCC

**Figure 6:**
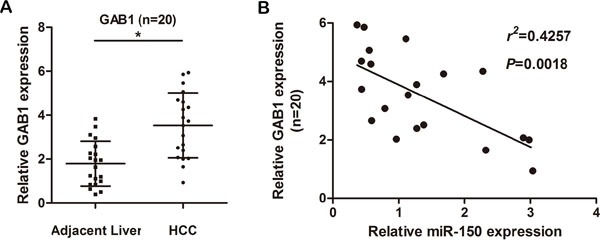
GAB1 levels are inversely correlated with miR-150 levels in HCC tissues **A.** GAB1 mRNA was detected in 20 HCC tissues and in matched adjacent noncancerous liver tissues by qRT-PCR. **B.** Spearman's correlation analysis between miR-150 levels and GAB1 mRNA levels in HCC tissues (r^2^=0.4257, *P*=0.0018). *P<0.05.

To further validate our finding that miR-150 can directly suppress GAB1 expression, we measured GAB1 expression in 20 human primary HCC tissues and adjacent noncancerous tissues and analyzed the relationship between GAB1 and miR-150 expression. As shown in Figure 6A, GAB1 mRNA expression was significantly higher in HCC tissues than in adjacent noncancerous tissues (*P*<0.05). Spearman's correlation analysis indicated a significant inverse correlation between miR-150 and GAB1 expression in the 20 HCC tissues examined (Figure 6B, *P*<0.05).

### miR-150 regulates the downstream signaling of GAB1

**Figure 7:**
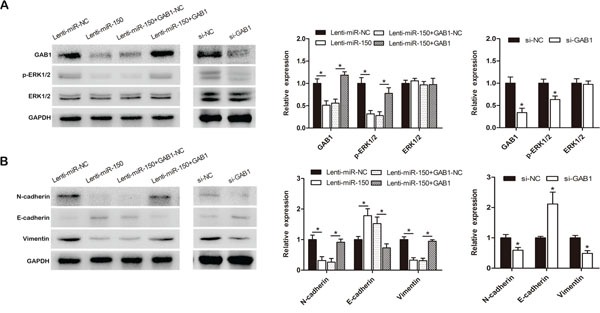
miR-150 exerts its functions by inhibiting the GAB1-ERK axis SMMC-7721 cells were transduced with Lenti-miR-150 or Lenti-miR-NC and transfected with pcDNA3.1-GAB1 or pcDNA3.1-GAB1-NC after stably overexpressing miR-150. The levels of GAB1, ERK1/2 and phospho-ERK1/2 (A, left) and of the EMT markers (E-cadherin, N-cadherin and Vimentin) (B, left) were evaluated by western blot analysis. SMMC-7721 cells were transfected with si-NC or si-GAB1. The levels of GAB1, ERK1/2 and phospho-ERK1/2 (A, right) and of the EMT markers (E-cadherin, N-cadherin and Vimentin) (B, right) were evaluated by western blot analysis. *P<0.05.

Previous studies reported that GAB1 is an adaptor protein that can mediate activation of the MAPK signaling pathway. To further investigate the mechanism responsible for the effects of miR-150, we measured the protein expression of representative markers of the MAPK pathway and EMT. miR-150 overexpression significantly decreased GAB1 and phospho-ERK1/2 protein expression compared to the control but did not alter total ERK1/2 protein levels (Figure 7A, left). Moreover, miR-150 overexpression enhanced the expression of an epithelial marker (E-cadherin) and reduced the expression of mesenchymal markers (N-cadherin and Vimentin) (Figure 7B, left). The effects of miR-150 on phospho-ERK1/2 and representative EMT markers were partially rescued by the ectopic expression of GAB1 (Figure 7A, 7B, left). Furthermore, the effects of GAB1 knockdown on phospho-ERK1/2 and EMT markers (E-cadherin, N-cadherin and Vimentin) were similar to the effects induced by miR-150 (Figure 7A, 7B, right).

To confirm the relationship between GAB1, phospho-ERK1/2 and miR-150, we further performed immunohistochemistry in tumors and lung metastatic nodules. Compared with the lesions from the MHCC97-H-miR-NC cell group, the lesions from tumors and lung metastatic nodules of the MHCC97-H-miR-150 group exhibited much weaker GAB1 and phospho-ERK1/2 staining, which is consistent with our *in vitro* results (Figure 3D, 3I).

## DISCUSSION

In this study, we demonstrated that miR-150 was significantly downregulated in HCC tissues and that decreased miR-150 expression was associated with worse clinicopathological characteristics and a poor prognosis. miR-150 overexpression inhibited the proliferation, migration and invasion of HCC cell lines *in vitro* and *in vivo*. Further experiments indicated that miR-150 directly targeted the GAB1-ERK axis to suppress cell proliferation and EMT, which inhibited the migration and invasion of HCC cells. An inverse correlation between miR-150 and GAB1 levels was found in HCC tissues. These results suggest that miR-150 may play an important role in the carcinogenesis of HCC.

Dysregulation of miRNAs occurs in many human tumors and alters various cell functions by affecting target gene expression. Previous reports have demonstrated that miR-150 is dysregulated in multiple types of cancers. This miRNA is downregulated in colorectal cancer [15], pancreatic cancer [22], liver cancer stem cells [23], chronic lymphocytic leukemia [24], advanced cutaneous T cell lymphoma [25], epithelial ovarian cancer [18], and esophageal squamous cell carcinoma [26], whereas it is upregulated in gastric cancer, lung cancer and breast cancer [19–21]. These studies have shown that miR-150 might perform diverse roles in different types of cancer, however, little is known about the role of miR-150 in liver cancer progression. We found that miR-150 is significantly downregulated in HCC tissues. Combined with the clinicopathological characteristics, the results showed that decreased expression of miR-150 in HCC tissues is associated with aggressive clinicopathological characteristics and a poor prognosis. The presence of decreased miR-150 expression in HCC samples is consistent with results from Li's group showing that miR-150-5p expression is downregulated in HCC tissues compared to paired non-tumor tissues [27]. We further investigated the relationship between miR-150 expression and the clinicopathological characteristics in HCC. To the best of our knowledge, this study is the first to analyze these results systematically. Our study indicated that miR-150 has potential as a novel biomarker for predicting and identifying the prognosis and progression of patients with HCC.

Tumor recurrence and metastasis are hallmarks of malignancy, which is an urgent problem for HCC therapy. In this study, we demonstrate that miR-150 play a crucial role in HCC by inhibiting cell proliferation, migration, invasion and metastasis both *in vitro* and *in vivo*. Furthermore, using a bioluminescent imaging system, we found that miR-150 significantly inhibited pulmonary metastasis in nude mice, which is consistent with our *in vitro* results and our clinicopathological analysis.

Many studies have been performed to address the issue of whether miR-150 has an effect on the development and progression of cancer. The specific targets of miR-150 vary across different malignancies. In pancreatic cancer cells, miR-150 was shown to bind to the 3′-UTR of c-Myb and MUC4 to regulate cell proliferation, migration and invasion [22]. In osteosarcoma, miR-150 functions as a tumor suppressor partially by targeting IGF2BP1 [28]. To investigate the role and underlying mechanisms of miR-150, we searched for miR-150 target genes in HCC. After performing bioinformatics analysis, we identified a sequence complementary to miR-150 in the 3′-UTR region of the GAB1 mRNA. miR-150 overexpression significantly downregulated both the mRNA and protein levels of GAB1 in HCC cells.

GAB1, which belongs to the Grb2-associated binder (Gab) family, functions as a scaffolding adaptor and is involved in tumorigenesis, invasion and metastasis [29–32]. In intrahepatic cholangiocarcinoma and hilar cholangiocarcinoma, GAB1 has been reported to promote cell proliferation and invasion and to decrease apoptosis [33, 34]. GAB1 expression is increased and strongly associated with tumor progression and prognosis in patients with HCC [35]. We found that GAB1 mRNA levels were inversely associated with miR-150 expression in HCC tissues, which suggested that GAB1 upregulation in HCC might be caused by miR-150 downregulation. Furthermore, GAB1 knockdown inhibited the growth, migration and invasion of HCC cells in a manner similar to miR-150 overexpression. Additionally, the inhibitory effects of miR-150 on HCC cells were partially reversed by the restoration of GAB1 expression. Taken together, these results indicate that GAB1 is a direct and functional target of miR-150 in HCC. Recently, Mraz's group found that the expression of GAB1 and FOXP1 is modulated by miR-150, resulting in proficient B-cell receptor signaling in chronic lymphocytic leukemia [24], which is consistent with our findings.

We further investigated the role and mechanism of the miR-150-GAB1 axis in HCC. GAB1 has been reported to act as a docking protein for several SH2-containing proteins and to coordinate signal transmission from receptors to downstream signaling pathways [30]. Upon stimulation, GAB1 activates the MAPK signaling pathway, which is important for regulating cell proliferation, migration and survival [29, 36]. Our study showed that miR-150 reduced phospho-ERK1/2 activation by downregulating GAB1. Recently, increasing evidence showed that induction of EMT of cancer cells correlates with the presence of vascular invasion and metastasis of HCC [37]. Both our group and other influential studies have demonstrated that phospho-ERK1/2 correlates with cancer-associated EMT [38–40]. Here, our study found that miR-150 overexpression inhibited EMT by reducing the phosphorylation of ERK1/2 in HCC cell lines. These results suggest that miR-150 may function as a tumor suppressor by inhibiting GAB1 protein expression and subsequent downstream ERK activation in HCC cell lines.

In conclusion, our study found that miR-150 was frequently downregulated in HCC and was associated with an aggressive tumor phenotype and a poor prognosis. miR-150 overexpression in HCC cell lines inhibited cell proliferation, migration and invasion *in vitro* as well as tumor growth and metastasis *in vivo*. The effects of miR-150 on HCC cell functions may be partially due to its regulation of GAB1 expression and subsequent downstream ERK activation. Taken together, these results demonstrate that miR-150 is a prognostic biomarker and that the miR-150-GAB1-ERK axis is a potential therapeutic target in HCC.

## MATERIALS AND METHODS

### Clinical specimen collection

Surgically resected HCC tissues and matched adjacent non-cancerous liver tissues were collected from 84 patients with HCC between 2005 and 2009 at the Department of Hepatobiliary Surgery, Xijing Hospital, the Fourth Military Medical University (Xi'an, China). None of the patients had received chemotherapy or radiotherapy before surgical resection. All specimens were immediately frozen in liquid nitrogen and stored at −80°C until analysis. The study was approved by the Ethics Committee of the Fourth Military Medical University. All participants were fully informed of the complete details of the study design and procedures. All participants or legal guardians of the participants gave written consent before inclusion in the study. All patients were followed for 5 years for survival calculations.

### Cell lines and cell culture

Human HCC cell lines MHCC97-H and SMMC-7721 were obtained from the Cell Bank of the Chinese Academy of Sciences (Shanghai, China). MHCC97-H cells labeled with luciferase (MHCC97-H-luc) were kindly provided by Prof. Yong Chen and Dr. Yang Cao from our laboratory. Cells were routinely cultured in Dulbecco's modified Eagle's medium (DMEM, HyClone, Logan, UT, USA) supplemented with 10% fetal bovine serum (FBS, Invitrogen, Carlsbad, CA, USA) in a humidified incubator containing 5% CO_2_ at 37°C.

### Plasmids and cell transfection

Lentiviral plasmids encoding miR-150 or negative control were designed and produced by Genechem (Shanghai, China). MHCC97-H and SMMC-7721 cells were grown in 6-well plates to 20–30% confluence, and the culture medium was replaced with transduction enhancing solution containing 30 MOI lentivirus and 50 μg/ml polybrene. After 12 h, the medium was replaced with complete medium, and the cells were cultured for 72 h. Then, the cells were selected with 1 μg/ml puromycin for 3 days and harvested for subsequent studies. HCC cells were transfected with miR-150 mimic, miR-150 mimic-NC (negative control), siRNA for GAB1 (si-GAB1-1, si-GAB1-2, si-GAB1-3), GAB1-siRNA-NC (si-NC) (GenePharma, Shanghai, China), pcDNA3.1-GAB1 plasmid lacking the 3′-UTR or pcDNA3.1-GAB1-NC (Genechem, Shanghai, China) using Lipofectamine 2000 (Invitrogen) in accordance with the manufacturer's instructions.

### Quantitative RT-PCR analysis

Total RNA was extracted from tissues or cultured cells with RNAiso Plus (TaKaRa, Dalian, China) for both miRNA and mRNA analysis. To determine the level of mature miR-150, we used a previously described stem-loop RT primer [41]. Total RNA was polyadenylated using a stem-loop-based First Strand Synthesis kit (Sangon Biotech, Shanghai, China) according to the manufacturer's product manual. Then, qRT-PCR was performed for miR-150 with a SYBR Premix Ex Taq miRNA kit (TaKaRa, Dalian, China) in accordance with the manufacturer's instructions. U6 small nuclear RNA was used as an internal control. To quantify GAB1 expression, qRT-PCR was performed using a SYBR Premix Ex Taq^TM^ II kit (TaKaRa, Dalian, China) in accordance with the manufacturer's instructions. β-actin was used as the reference gene. The relative fold changes in the levels of GAB1 and miR-150 were calculated using the 2^−ΔΔCT^ method. All measurements were performed three times. The primer sequences are shown in Supplementary Table 1.

### Western blot analysis

Whole cells or tissue extracts were harvested in RIPA lysis buffer (Beyotime, Shanghai, China) containing protease inhibitors (Thermo Scientific, Rockford, IL, USA) and phosphatase inhibitors (Thermo Scientific). The proteins were quantified using a BCA protein assay kit (Thermo Scientific) according to the manufacturer's instructions. Total protein extracts were separated by 10% SDS-PAGE and transferred to polyvinylidene difluoride membranes. The membranes were incubated with primary antibodies, including anti-GAB1 (Millipore, Billerica, MA, USA), anti-ERK1/2 (Cell Signaling Technology CST, Danvers, MA, USA), anti-p-ERK1/2 (CST), anti-N-cadherin (CST), anti-E-cadherin (CST), anti-Vimentin (CST) and anti-GAPDH (Abcam, Cambridge, UK), overnight at 4°C. After the membranes were incubated with horseradish peroxidase (HRP)-conjugated secondary antibodies (Abcam) for 1 h at room temperature, the protein bands were detected with a ChemiDoc^TM^ XRS+ and Image Lab TM software (Bio-Rad, Hercules, CA, USA).

### Cell proliferation and colony formation assays

For the CCK8 assay, transfected MHCC97-H cells or SMMC-7721 cells were seeded in 96-well plates, and cell proliferation was measured at 1, 2, 3, 4 and 5 days by Cell Counting Kit 8 (CCK-8, Yiyuan, Guangzhou, China) assay according to the manufacturer's instructions. Each experiment included six replicates and was repeated three times. For the colony formation assay, 300 transfected HCC cells were seeded in a 6-well plate and cultured for 14 days in DMEM supplemented with 10% FBS. Then, the colonies were fixed in 95% ethanol and stained with a 4 g/L crystal violet solution. Colonies containing over 50 cells were counted.

### Cell migration and invasion assays

Cell migration and invasion were detected using Transwell chambers (8 μm pore size; Millipore) with (invasion assay) or without (migration assay) Matrigel (BD Biosciences, San Jose, CA, USA) matrix. In brief, 600 μl complete medium was added to the bottom chamber, transfected cells were suspended in serum-free medium, and 200 μl of the cell suspension (containing 4×10^4^ cells) was placed in the upper chamber. After 24 hours, the cells on the top surface of the membrane were mechanically removed using a cotton swab, and the cells on the bottom surface of the membrane were fixed in 95% ethanol and stained with a 4 g/L crystal violet solution. Cells adhering to the bottom surface of the membrane were counted in five randomly selected areas under a 200× microscope field. Each experiment was repeated three times.

### *In vivo* tumor growth and metastasis experiments

For the tumorigenesis assay, transfected MHCC97-H cells (2×10^6^) were suspended in 150 μl PBS and subcutaneously injected into the left flank of nude mice (n=5 mice per group). Tumors were measured with a digital caliper every 7 days, and the tumor volume was calculated by the following formula: tumor volume=(length×width^2^)/2. Tumors were harvested and weighed after the mice were euthanized at the end of the experiment. The dissected tumors were frozen in liquid nitrogen or fixed in formalin and paraffin-embedded for H&E staining and immunohistochemistry.

For the metastasis assay, transfected MHCC97-H-luc cells (2×10^6^) suspending in 150 μl PBS were injected into nude mice through the tail vein (n=6 mice per group). After 35 days, the mice were anesthetized and intraperitoneally injected with 150 μg/body weight (g) of D-luciferin (Caliper, Hopkinton, MA, USA). Fifteen minutes later, the bioluminescence from each mouse was imaged in an IVIS Lumina II Imaging System (Caliper). Afterward, the mice were euthanized, and the lungs were removed, fixed in formalin and paraffin-embedded for H&E staining and immunohistochemistry.

Nude mice (BALB/C nu/nu, 5-week-old, female) were obtained from the Animal Center of the Chinese Academy of Science (Shanghai, China) and maintained under specific pathogen-free conditions in the laboratory animal center of Fourth Military Medical University (Xi'an, China). All experimental procedures involving mice were performed in accordance with the Guide for the Care and Use of Laboratory Animals and were approved by The Research Animal Care and Use Committee of Fourth Military Medical University.

### Immunohistochemistry

Tissue samples were fixed in formalin, embedded in paraffin and sectioned to 4-μm thickness. The sections were deparaffinized, hydrated, and boiled in 10 mM citrate buffer (pH 6.0) for antigen retrieval. Endogenous peroxidases were inactivated with 3% H_2_O_2_. After the sections were blocked in goat serum, the sections were incubated with primary antibody (Ki-67, Boster, Wuhan, China; GAB1, Millipore; p-ERK1/2, CST) overnight at 4°C, incubated with biotinylated secondary antibody at room temperature for 1 h, and visualized with diaminobenzidine (DAB kit, ZSGB-BIO, China). Hematoxylin was used to counterstain the nuclei.

### Luciferase reporter assay

Cells were seeded in a 24-well plate and co-transfected with luciferase reporter constructs encoding wild-type 3′-UTR region of GAB1 (GAB1-WT-3′-UTR) or a mutated GAB1 3′-UTR region (GAB1-MUT-3′-UTR) (RiboBio, Guangzhou, China) and miR-150 mimic or miR-150 mimic-NC using Lipofectamine 2000 (Invitrogen). After the cells were incubated for 48 h, they were washed with PBS and lysed with Passive Lysis Buffer (Promega). Firefly and *Renilla* luciferase activities were measured using a Dual-Luciferase Reporter Assay Kit (Promega) according to the manufacturer's protocol. All experiments were repeated three times.

### Statistical analysis

All data are presented as the mean±SD. The differences between means were analyzed with a t-test. The differences in the clinical pathological characteristics between the two miR-150 groups were analyzed by χ2 test. Survival curves were calculated using the Kaplan-Meier method and compared by log-rank test. Cox proportional hazards analysis was used for univariate and multivariate analyses to assess the effect of miR-150 and clinical pathological characteristics on survival. The relationship between GAB1 and miR-150 was explored by Spearman's correlation. All statistical analyses were performed using SPSS version 17.0 (IBM, Chicago, IL, USA), and all figures were generated using GraphPad Prism 5.01 (GraphPad Software, La Jolla, CA, USA). *P*<0.05 were considered statistically significant.

## SUPPLEMENTARY FIGURES AND TABLE


