# Supplementary material for: MicroRNA-150 suppresses cell proliferation and metastasis in hepatocellular carcinoma by inhibiting the GAB1-ERK axis
Source: Oncotarget. 2016 Feb 9;7(10):11595–608. doi: 10.18632/oncotarget.7292 (PMC4905496; doi:10.18632/oncotarget.7292)
Supplement: Supplementary file 1 [file oncotarget-07-11595-s001.pdf]

SUPPLEMENTARY FIGURES AND TABLE

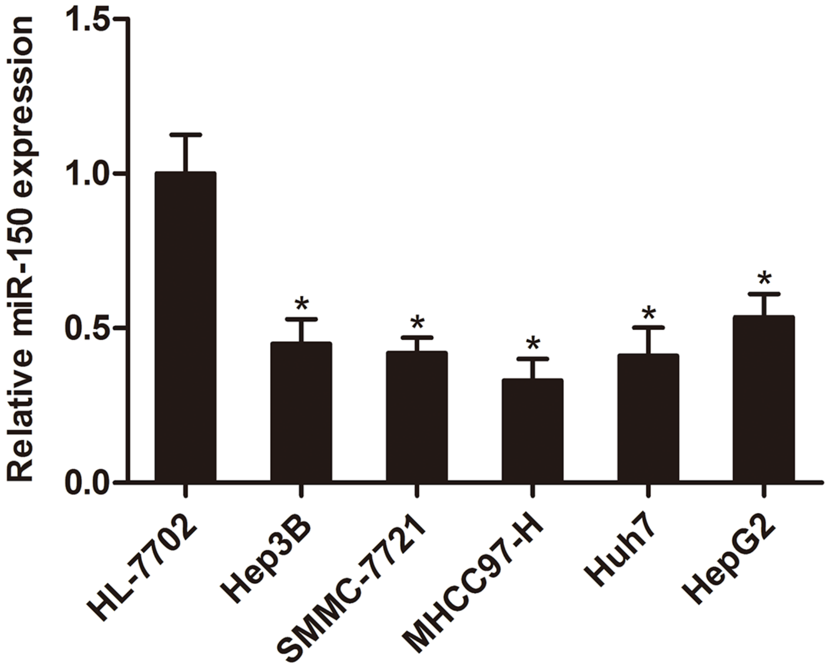

Supplementary Figure 1: miR-150 is significantly downregulated in HCC cell lines. Relative expression levels of miR-150 in a normal human hepatocyte cell line (HL-7702) and HCC cell lines (Hep3B, SMMC-7721, MHCC97-H, Huh7, HepG2).*P<0.05.

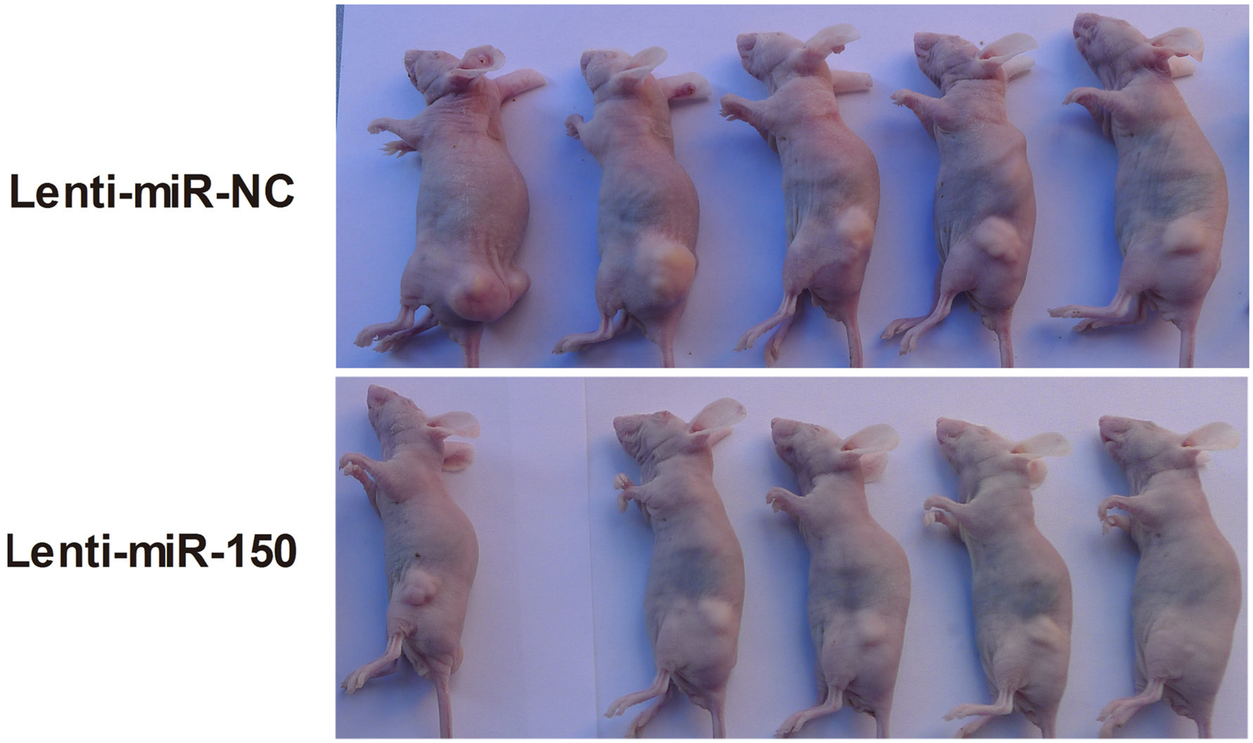

Supplementary Figure 2: miR-150 suppresses tumor growth *in vivo*. Transduced MHCC97-H cells were injected subcutaneously into nude mice (n=5). After 28 days, the mice were euthanized, and the tumors were excised. All the mice with implanted HCC cells are shown.

Supplementary Table 1: Primer sets used for qRT-PCR

Primer	Sequence
miR-150-RT	5'-gtcgtatccagtgcagggtccgaggtattcgcactggatacgacCACTGG-3'
miR-150- Forward	5'-tcggcgTCTCCCAACCCTTGTAC-3'
miR-150- Reverse	5'-GTCGTATCCAGTGCAGGGTCCGAGGT-3'
U6-RT	5'-AAAATATGGAACGCTTCACGAATTTG-3'
U6- Forward	5'-CTCGCTTCGGCAGCACATATACT-3'
U6- Reverse	5'-ACGCTTCACGAATTTGCGTGTC-3'
GAB1- Forward	5'-GATGGTTCGTGTTACGCAGTG-3'
GAB1- Reverse	5'-CGCTGTCTGCTACCAAGTAGAA-3'
β-actin- Forward	5'-TAGTTGCGTTACACCCTTTCTTG-3'
β-actin- Reverse	5'-TCACCTTCACCGTTCCAGTTT-3'
